# Lithium Battery Ingestion: An Unusual Cause of Bilateral Cord Palsy

**DOI:** 10.1155/2015/790830

**Published:** 2015-09-17

**Authors:** Gautam Bir Singh, Ravinder Chauhan, Deepak Kumar, Rubeena Arora, Shruti Ranjan

**Affiliations:** Department of Otorhinolaryngology, Head & Neck Surgery, Lady Hardinge Medical College & Associated Hospitals, Shaheed Bhagat Singh Marg, New Delhi 110001, India

## Abstract

Bilateral vocal cord palsy is a rare but life threatening complication of lithium battery ingestion in children. This complication is mostly missed by otorhinolaryngologists due to lack of awareness on the cited subject. We present one such rare case in an infant, where the clinical presentation was found to be unique but hitherto unreported in the medical literature. This clinical record discusses this case in light of the scant current medical literature on the subject and highlights the importance of cautious monitoring of patients presenting with signs of respiratory distress after lithium battery removal.

## 1. Introduction

The incidence of lithium battery (LB) ingestion has been increasing in the urban areas worldwide over a period of time [1, 2]. Ingested lithium batteries are associated with significant morbidity and occasional mortality if they get impacted in oesophagus [2]. They may cause dreadful late complications like oesophageal perforation, tracheoesophageal fistulas, exsanguinations after fistulisation into major blood vessels, oesophageal stricture, and vocal cord palsies [2–4]. Thirteen deaths attributed to LB ingestion have been reported in medical literature [3]. Recent review of literature cites bilateral vocal cord (BLVC) palsy as yet another life threatening complication of LB ingestion. However, there is a marked paucity of literature on the cited subject, with only 4 reported cases in the English medical Literature till date [4–7]. We herein report a case of this rare entity which presented in an extremely rare manner.

## 2. Case Report

**Figure 1:**
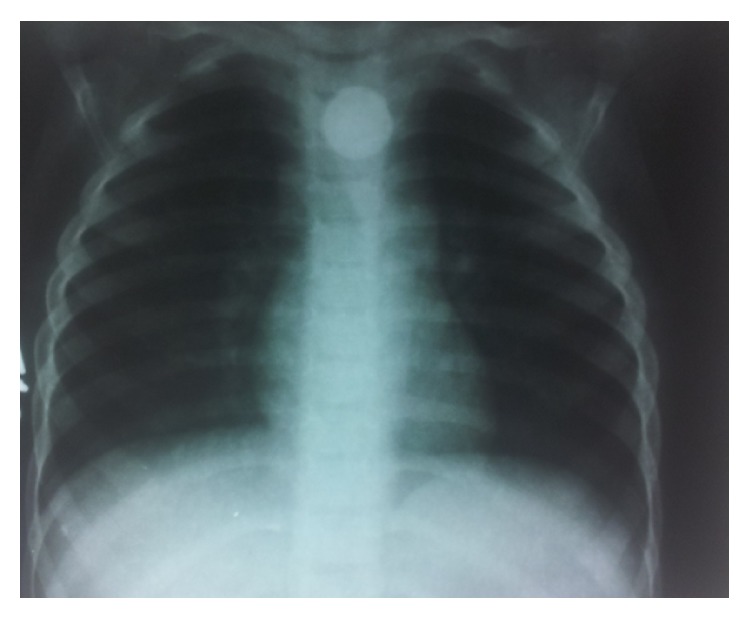
X-ray chest PA view showing a radio-opaque foreign body at the level of cricopharynx.

**Figure 2:**
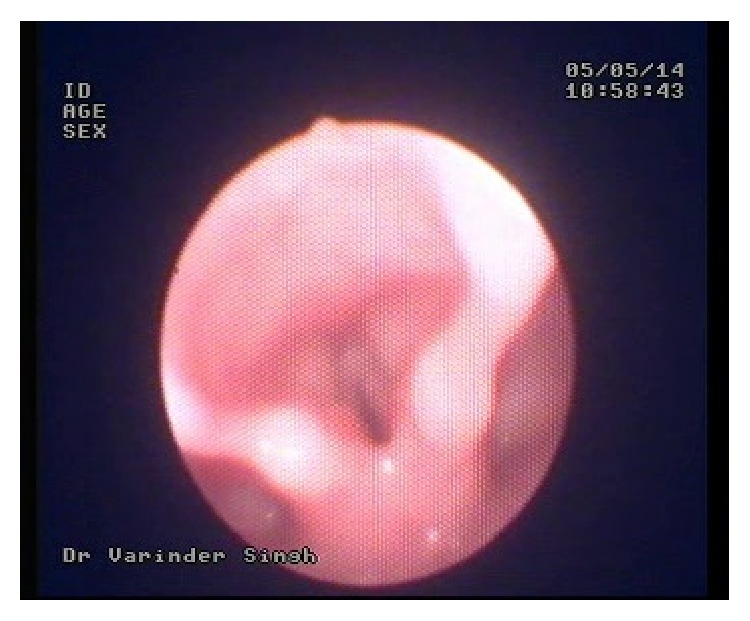
Clinical photograph showing paralyzed true vocal cords in paramedian position just after tracheostomy.

**Figure 3:**
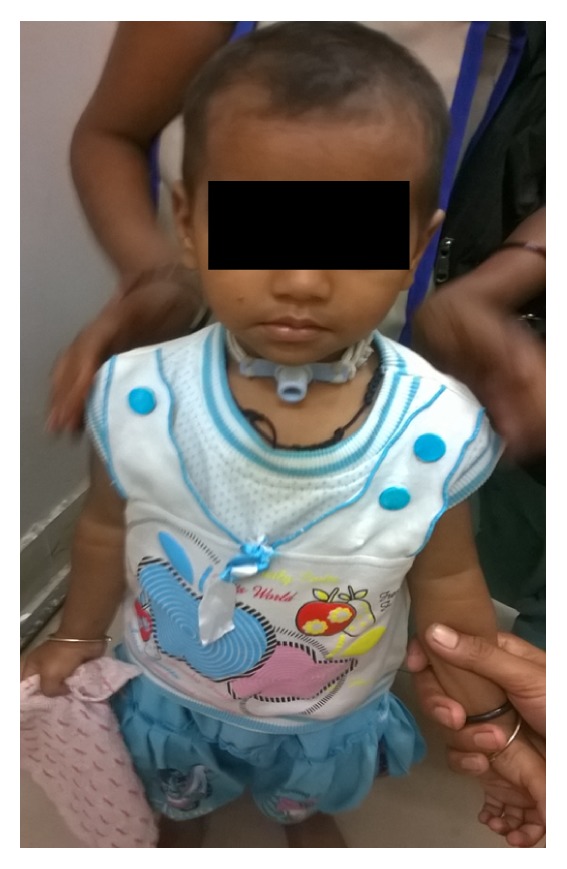
Clinical photograph of the patient at 6-month follow-up.

A 10-month-old baby girl reported to our tertiary care teaching hospital with the complaint of stridor for the past 1 day. The baby was not accepting feeds and also had drooling of saliva. A routine X-ray chest to rule out chest infection revealed a characteristic well delineated double radio opaque shadow just below the cricopharynx (Figure 1). A diagnosis of unwitnessed lithium battery ingestion was made and the said foreign body was removed endoscopically under general anaesthesia. It would be pertinent to note that at the time of removal marked charring with slough was seen in the cricopharynx region. Postoperatively, a dramatic improvement was seen in the patient's condition. Subsequently patient was discharged on the 7th postoperative day with no untoward complaints. However, within a week of discharge, patient developed mild stridor on exertion (e.g., while running and playing), which gradually worsened over a period of time. A paediatric referral was sought and the patient was treated for tracheobronchitis, following which, her symptoms showed some improvement. However, two months after the removal of the foreign body; patient presented in the emergency with acute stridor. An immediate tracheostomy was done as all attempts to intubate the child failed. Later, endoscopic examination of vocal cords revealed that the patient was having bilateral vocal cord palsy (Figure 2). The patient is in regular follow-up with us (Figure 3). However for the last 6 months there has been no improvement in the vocal cord status. The parents have been counselled for surgical treatment of the patient if no recovery occurs within the next 6 months.

## 3. Discussion

A brief review of the salient features of all the previously reported 4 cases and this case of BLVC palsy following LB ingestion is presented in Table 1. It is important to note that even after expeditious removal of LB, the complication of BLVC palsy was recorded in these cases. It is imperative to note that lithium battery impaction in oesophagus is a grave medical emergency as untoward effects have been reported in duration as short as 2 to 8 hours [2–4]. Moreover, as most of the cases of LB ingestion are unwitnessed, the exact duration of impaction may be misleading, which may further complicate the matters. In addition, even after removal of LB, toxicity can persist and cause delayed complications [4]. A LB causes BLVC palsy by directly injuring the nerves due to lithium infiltration in the surrounding tissue. The most likely cause for lithium induced battery injury is generation of external current that causes electrolysis of tissue fluids, generating hydroxide ions at the battery's negative pole [2–4].

Bilateral vocal cord palsy is a life threatening complication which always presents with acute respiratory distress. All the previous cases required assisted ventilation, and some failed extubation (Table 1). Interestingly, our case had no respiratory distress at the time of discharge, but over a period of time the baby gradually developed features of respiratory obstruction which finally culminated in form of acute respiratory distress warranting tracheostomy. It would be prudent to note that in BLVC palsy the vocal cords may not drift to the midline immediately after neural injury but may take days or weeks to do so and thus cause airway compromise later as happened in our case. Hence a patient may present with mild stridor on exertion to life threatening airway obstruction [8]. This patient presented with the subclinical features of bilateral vocal cord palsy, but the diagnosis was missed over the period of 2-month follow-up. In this context it is interesting to note that in all such previously reported cases, the diagnosis of BLVC palsy was missed, and patients were diagnosed later on after initial management of airway (time varying from 3 to 28 days, Table 1). However, none of the authors have focussed on this aspect of diagnosis. We thus believe that (i) simple recording of vocal cord movements at the time of extubation by the operating surgeon during removal of LB, especially if there is necrosis at the site of impaction, (ii) and an active follow-up with close monitoring even after judicious removal of foreign body for detection of early signs of respiratory distress would go a long way in diagnosing this life threatening complication and help us to formulate a better management protocol in the best interest of patient care.

The treatment protocol for BLVC palsy dictates that these cases should be kept under observation for at least 1 year after initial airway management as spontaneous recovery does occur in such cases [9]. Only if this spontaneous recovery fails, surgery for decannulation should be considered.

In summary, this report raises the awareness among clinicians as to the various clinical presentations of this rare complication of LB. Hence, BLVC palsy should be considered as an important differential in all cases of LB removal postoperatively presenting with signs and symptoms of respiratory distress. This clinical record also underlines the importance of vocal cord examination at the time of endoscopic removal of LB in diagnosis of this life threatening complication.

## Figures and Tables

**Table 1:** Salient features of the cases with bilateral vocal cord palsy following lithium battery ingestion.

Authors/year	Age/sex	Time before LB removal	Endoscopic findings at the time of removal	Observation of bilateral vocal cord palsy	Presentation of BLVC palsy in postoperative period	Postoperative course
(1) Nagao et al. (2007) [7]	8 yrs/M	2 h	Erosion of postcricoids area	At day 12	Respiratory distress	No complication
(2) Bernstein et al. (2007) [6]	11 mth/F	5 h	Erosion of ant and lateral walls of hypopharynx	At day 6	Respiratory distress	Stridor with enteral feeding
(3) Hamilton et al. (2009) [5]	9 mth/F	NR	Erosion of the esophagus	At day 28	Respiratory distress	Supraglottoplasty with enteral feeding
(4) Simonin et al. (2013) [4]	16 mth/M	48 h	Erosion of oesophageal mucosa	At day 3	Respiratory distress	Posterior cordotomy with enteral feeding
(5) This case (2014)	10 mth/M	48 h	Erosion at the cricopharynx area	At day 60	Delayed respiratory distress	Tracheostomy

Key: BLVC: bilateral vocal cord palsy, yrs: years, mth: months, M: male, F: female, h: hours, and NR: not recorded.
